# Comparative phylogeny and evolutionary analysis of Dicer-like protein family in two plant monophyletic lineages

**DOI:** 10.1186/s43141-022-00380-x

**Published:** 2022-07-12

**Authors:** Behzad Hajieghrari, Naser Farrokhi, Mojahed Kamalizadeh

**Affiliations:** 1 Department of Agricultural Biotechnology, College of Agriculture, Jahrom University, Jahrom, Iran; 2 Department of Cell & Molecular Biology, Faculty of Life Sciences & Biotechnology, Shahid Beheshti University, Evin, Tehran, Iran

**Keywords:** Eudicotyledons, Evolutionary history, Functional domain, Liliopsida, Phylogenetic analysis

## Abstract

**Background:**

Small RNAs (sRNAs) that do not get untranslated into proteins exhibit a pivotal role in the expression regulation of their cognate gene(s) in almost all eukaryotic lineages, including plants. Hitherto, numerous protein families such as Dicer, a unique class of Ribonuclease III, have been reported to be involved in sRNAs processing pathways and silencing. In this study, we aimed to investigate the phylogenetic relationship and evolutionary history of the DCL protein family.

**Results:**

Our results illustrated the DCL family of proteins grouped into four main subfamilies (DCLs 1–4) presented in either Eudicotyledons or Liliopsids. The accurate observation of the phylogenetic trees supports the independent expansion of DCL proteins among the Eudicotyledons and Liliopsids species. They share the common origin, and the main duplication events for the formation of the DCL subfamilies occurred before the Eudicotyledons/Liliopsids split from their ancestral DCL. In addition, shreds of evidence revealed that the divergence happened when multicellularization started and since the need for complex gene regulation considered being a necessity by organisms. At that time, they have evolved independently among the monophyletic lineages. The other finding was that the combination of DCL protein subfamilies bears several highly conserved functional domains in plant species that originated from their ancestor architecture. The conservation of these domains happens to be both lineage-specific and inter lineage-specific.

**Conclusions:**

DCL subfamilies (i.e., DCL1-DCL4) distribute in their single clades after diverging from their common ancestor and before emerging into higher plants. Therefore, it seems that the main duplication events for the formation of the DCL subfamilies occurred before the Eudicotyledons/Liliopsida split and before the appearance of moss, and after the single-cell green algae. We also observed the same trends among the main DCL subfamilies from functional unit composition and architecture. Despite the long evolutionary course from the divergence of Liliopsida lineage from the Eudicotyledons, a significant diversifying force to domain composition and orientation was absent. The results of this study provide a deeper insight into DCL protein evolutionary history and possible sequence and structural relationships between DCL protein subfamilies in the main higher plant monophyletic lineages; i.e., Eudicotyledons and Liliopsida.

**Supplementary Information:**

The online version contains supplementary material available at 10.1186/s43141-022-00380-x.

## Background

Small RNAs (sRNAs) are 20–30 nucleotides long belonging to the distinct class of the large group of RNAs transcribed from genomic DNA but not translated into proteins [4, 14]. sRNA-mediated gene regulation is one of the fundamental molecular mechanisms having a pivotal role in the regulation of their cognate gene(s) expression in a locus-specific manner at both transcriptional level by DNA methylation and posttranscriptional level via mRNA cleavage and, or translational inhibition [14, 40]. sRNAs and their processing pathways are conserved and have distributed in almost all eukaryotes [15, 23, 46]. Such regulatory mechanisms are essential for fine-tuning the expression of the corresponding genes [25]. The relatively processing of small RNAs and their transcript silencing process is a complex system [6, 10, 14], which prompts comprehensive understanding of its components an absolute necessity. Dicer (DCL), a unique class of Ribonuclease III (RNase III) family of enzymes that is one such component, interacts with several associated proteins in the processing of small RNA precursors [12, 43]. It exhibits a key role in processing long double-stranded RNA substrates into uniformly sized small RNA(s) with 2-nucleotide overhangs at the 3′-ends [27, 42].

Plant dicer protein is a large multi-domain (six domains: DExH Helicase, DUF283, PAZ, RNase IIIa, RNase IIIb, and dsRNA binding (dsRB) domain) protein as delineated by its crystal structure [12, 28]. One or more occupations may be eliminated or absent from the final folding [28]. The double-stranded RNA-binding (dsRBD) domain recognizes and binds to dsRNA in a non-specific manner [31]. The C-terminus of dsRBDs can interact with protein rather than dsRNA to pair with DCL proteins [5]. The PAZ domain is directly connected to the RNaseIIIa domain by a long α-helix. It can recognize and bind to two overhang bases at the 3′-end of the dsRNA precursor. It is also interesting to consider that the PAZ domain can bind single-stranded RNAs [21]. The two RNase III domains provide the main catalytic activity, cut dsRNA precursor to release short RNA duplexes with 2-nucleotide overhangs at the 3’-end and phosphorylated 5′-ends [5, 21]. It argues that the distance between the PAZ and RNaseIII domain determines the length of the cleaved sRNA, and it is considered the source of mature sRNA length variants [48].

Plants evolutionary have expanded the number of their DCLs: four in Arabidopsis (DCL1, DCL2, DCL3, and DCL4) and six in *Medicago truncatula* [29, 34]. The homologs enzymes produce mature small RNAs with distinct sizes and regulatory speciation [30]. In *A. thaliana*, DCL1 and DCL4 yield 21 nt, DCL2 generate 22 nt, and DCL3 creates 24 nt [37]. Apart from the length, miRNA genes are formed by DCL1 [2, 45]. They are involved in producing functional small RNAs from endogenous inverted repeats. However, DCL2 has a significant role in generating small RNAs from natural cis-acting antisense transcripts. DCL3 performs a direct role in creating 24 nt-long small RNAs related to site-specific DNA methylation and chromatin modification. DCL4 remains a critical component in the formation of ta-siRNA and performing post-transcriptional silencing [26]. DCL proteins are essential either in eukaryotic growth or in development. They are responsible for defending the cell against invading gene creatures, including but not limited to viruses and active transposable elements [16, 41]. For the latter, DCL2 and DCL4 are the essential players in viral genome duplication and systemic infiltration in plants [35]. A convincing piece of evidence suggests that the DCL gene family originated early in the Eukaryote evolution right at the time of multicellularization and then expanded in the corresponding kingdoms [33]. In plants, DCL homologs diverged before the appearance of moss Physcomitrella patens and after the single-cell green algae *Chlamydomonas reinhardtii* [26].

However, many reports involved in the functional role of the Dicer proteins in the processing of non-coding RNAs, their evolution is still in its infancy. In this study, we investigated the pattern of plant Dicer evolutionary history and possible relationships between DCL protein families in the main plant monophyletic lineages via protein sequence analyses and conserved motifs composition, phylogenetic tree reconstruction, evolutionary history inference, and functional domain identification and architecture.

## Methods

### Data collection

Sequences stored as DCL protein for Liliopsida and Eudicotyledons plant species were isolated using a keyword search, “Dicer-like protein (DCL)” in a non-redundant protein database (http://www.ncbi.nlm.nih.gov). Sequences were retrieved and stored in FASTA format. We removed duplicated and partial sequences in each plant species using Clustal Omega and CodonCode v.8.0.2 aligner tools. Additionally, we checked the structure, protein domain families, and function of all proteins using Uniprot, Pfam, and SMART databases and removed redundant sequences. We employed 274 full-length Dicer-like proteins from Liliopsids and Eudicotyledons families for further analysis.

### Multiple sequence alignment (MSA)

We constructed multiple protein sequence alignments (MSAs) using MAFT [20], MUSCLE [9], Kalign [22], T-Coffee [8], and Clustal Omega [39] with their default parameters. To measure the quality of the alignments and gauge the performance of the algorithms in aligning the data sets, we computed the sum-of-pair score (SP-score), the column score (C-score), and transitive consistency score (TCS-score) of the produced alignment. Then we evaluated the relative reliability of constructed MSAs for each data set using finding the best amino acid substitution model and calculating the maximum log-likelihood. MSA with the lowest Bayesian Information Criterion (BIC) score and maximum log-likelihood nearest to zero taken as the best structurally correct sequences alignment for further analysis. The alignment file was visualized and analyzed using the BioEdit sequence alignment editor [17].

### Protein primary sequence features

The protein primary sequence features were determined using the Molecular Evolutionary Genetics Analysis software (MEGA) 11.0.10 [44]. The amino acids frequencies were predicted in the DCL sequences set. We specified the best amino acid substitution pattern for the specific sequences and selected the successful model with the most negative BIC scores (Bayesian Information Criterion) for the amino acid substitution matrix. The evolutionary divergence estimated between each possible pair of sequences typically uses the best-fitting amino acid substitution model. Additionally, we used the selected substitution model to compute the number of amino acid substitutions per site from each pair of sequences and overall sequences.

### Motif and domain prediction

We used the Multiple EM for Motif Elicitation (MEME; http://memesuite.org/ [1]) to identify protein sequences containing motifs. The following parameters were set: (1) each motif site assigned zero or one occurrence per sequence; (2) optimum motif widths were between 6 and 50; (3) the maximum number of motifs was 20. Moreover, we employed ScanProsite (https://prosite.expasy.org/scanprosite/ [7]) for predicting conserved domains of DCL protein sequences. Then we aligned the identified motif sequences from each DCL proteins sets and compared them within the Liliopsida and Eudicotyledons species. The functional domains of the predicted motifs and their composition for each DCL protein sequence were identified using Pfam (http://pfam.janelia.org/ [32]) database and confirmed each inferred domain using the SMART (http://smart.embl-heidelberg.de/smart/set_mode.cgi?NORMAL=1 [38]) database.

Proteins are composed of multiple functional units of common descent, and comparing domain composition and architecture is a beneficial method for the evolutionary analysis of homologous proteins. In this sense, the arrangement and the order of the DCL protein domains on its primary sequence, queried from Pfam, were determined via the prediction of functional units at the Hmmscan search tool (https://www.ebi.ac.uk/Tools/hmmer/search/hmmscan [11]).

### Phylogenetic analysis

For phylogenetic analysis of the DCL protein family in plant species, we constructed the unrooted tree based on maximum likelihood (ML) heuristic methods in MEGAX 11.0.10 [44]. We employed aligned sequences of the plant DCLs under the selected model for the substitution-rate matrix. Bootstrapping was performed with 500 replicates. Then we rooted the tree using an outgroup, DCL from *Auxenochlorella protothecoides*. The rooted tree represents the last common ancestor of all groups in the tree by directing evolutionary time. The trees were displayed using the iTOL v5 online tool [24].

### Comparative analyses of protein structure

HHpred server (https://toolkit.tuebingen.mpg.de/tools/hhpred [50]) accessible in Toolkit (https://toolkit.tuebingen.mpg.de/ [13]) was used to search a significant match with a protein of known structure in the PDB database. We employed the MODELLER to build the atomic coordinates of the proteins and create a structural file in PDB [47]. In addition, we used the DALI server (http://ekhidna2.biocenter.helsinki.fi/dali/ [18]) for structural comparison and visualization superimposition of the predicted models. Dali scores (Dali *Z*-scores) are used to establish structural similarity and relationships between proteins resulting from the dendrogram constructed by an average linkage clustering of the structural similarity matrix. Root mean square deviation (RMSD), which measures the deviation between two superimposed atomic coordinates, were compared among the encoded DCL subfamily structures of *A. thaliana*. The Ramachandran plot (https://zlab.umassmed.edu/bu/rama/) was employed to compare the allowed regions of conformational space available to the protein chains by uploading the PDB-predicted file.

## Results and disscusion

### MSA and DCL sequence characteristics

After discarding the redundant protein sequences obtained from the non-redundant protein database at NCBI, we collected 31 and 242 DCL candidate protein sequences from 13 and 60 Liliopsida and Eudicotyledons species, respectively (Supplementary file 1). From the constructed MSAs (Table 1), Muscle-based MSA resulted in the highest maximum log-likelihood trees and was considered the most reliable algorithm (Supplementary file 2) for phylogenetic and evolutionary analysis. We computed the Jones-Taylor-Thornton (JTT) +G+I+F model for the Liliopsids protein set and JTT + G for the Eudicotyledons proteins. These representations obtain the most negative BIC scores (61542.366184322 and 31230.133303795) for the series of the aligned sequence, respectively. Therefore, they considered the best in describing the substitution pattern in these sets (Table 1). In addition, the discrete gamma distribution was estimated under these models to be 0.9976 and 1.2493 separately.

**Table 1:** The quality of constructed multiple sequence alignment (MSA) with the algorithms based on the sum-of-pair score (SP-score), the column score (C-score), and the Transitive Consistency Score (TCS-score), and the predicted best amino acid substitution model based on the lowest BIC^a^ score (A) and the calculated maximum log-likelihood for constructed tree from each MSA set (B)

**Alignment algorithm**	**Liliopsida lineage**						**Eudicotyledons lineage**					
	**SP-score**	**TC-score**	**TCS-score**	***A***	BIC^*^ scores	B**	**SP-score**	**TC-score**	**TCS-score**	***A***	**BIC scores**	***B***
**T-coffee**	326748	108	891	JTT + G + I + F***	61968.7167205287	− 30581.59	17432842	5	883	JTT + G	31409.2078965051	− 13324.74
**MUSCLE**	328078	101	881	JTT + G + I + F	61542.366184322	− 30401.81	17489758	11	866	JTT + G	31230.133303795	− 13347.87
**Kalign**	327823	133	875	JTT + G + I + F	63956.652705501	− 31582.69	17301996	5	836	JTT + G	40832.5711781908	− 18038.05
**Clustal omega**	324620	110	855	JTT + G + I + F	62178.742941649	− 30694.67	17217684	7	854	JTT + G	32693.3283402443	− 14250.52
**MAFT**	328316	112	885	JTT + G + I + F	62421.8477405952	− 30804.25	17619344	13	883	JTT + G	33692.6193749731	− 14453.60

***JTT Jones-Taylor-Thornton, *G* discrete Gamma distribution, *I* a certain fraction of sites is evolutionarily invariable, *F* amino acid frequencies

**The tree was inferred by Neighbor-Joining method

*BIC Bayesian Information Criterion

### Phylogenetic tree reconstruction and evolutionary analysis

**Fig. 1:**
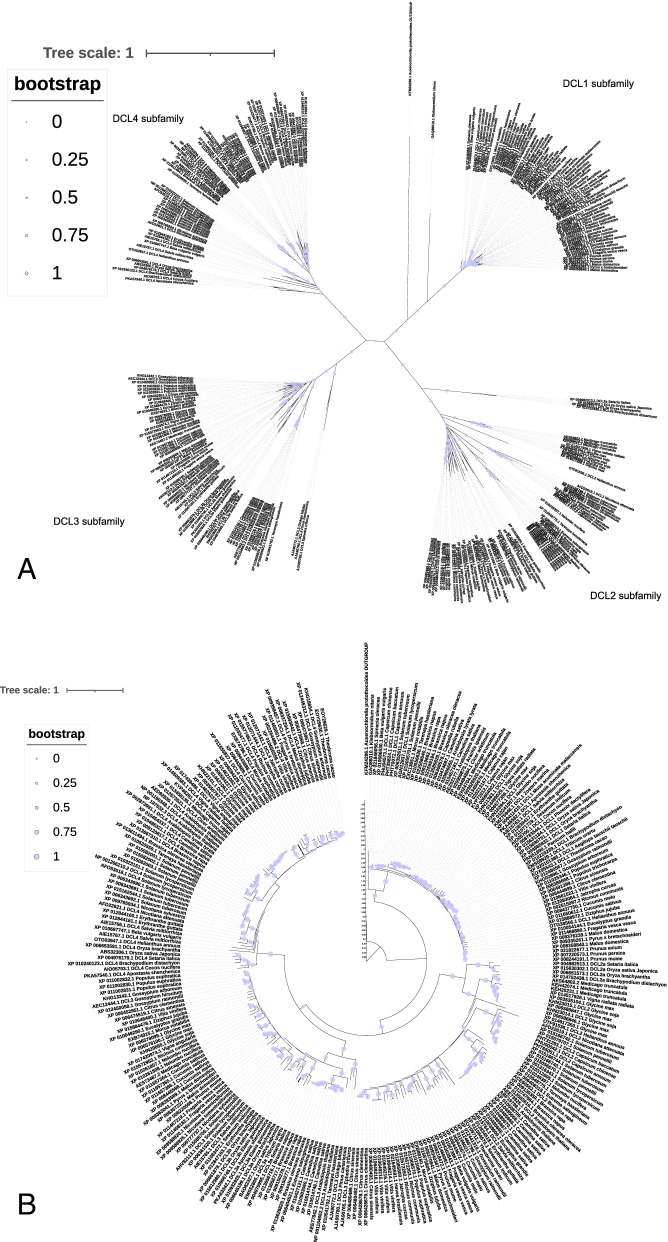
**A:** Unrooted phylogenetic tree of the DCL protein related to higher plants. **B:** The tree was rooted with the outlier (outgroup *A. protothecoides* DCL protein). The Phylogenetic relationship was inferred from full-length polypeptide sequences of the plant DCL proteins using the Maximum Likelihood method and JTT model [19] with log likelihood of − 18013.10. The percentage of trees in which the associated taxa clustered together in the bootstrap test (500 replicates) is shown as a symbol displayed on each branch (Felsenstein, 1985). Initial tree(s) for the heuristic search were obtained automatically by applying Neighbor-Joining and BioNJ algorithms to a matrix of pairwise distances estimated using the JTT model. Topology with superior log likelihood value was selected. A discrete gamma distribution was used to model evolutionary rate differences among sites (2 categories (+*G*, parameter = 1.6882)). The rate variation model allowed some sites to be evolutionarily invariable ([+*I*], 1.53% sites). The tree is drawn to scale, with branch lengths measured in the number of substitutions per site. This analysis involved 275 polypeptide sequences from Eudicotyledons (242) and Liliopsida (31) and *Klebsormidium nitens* and *A. protothecoides* DCL polypeptide sequences used as outlier. All positions containing gaps and missing data were eliminated (complete deletion option). 131 positions in the final dataset was seen. Evolutionary analyses were conducted in MEGA11 and visualized by iTOL v5 online tool [24]

DCL proteins formed an expanding family across different plant lineages. To deepen our understanding of how the DCL protein family evolved and know the evolutionary relatedness of the DCL proteins in the plant lineages, we constructed their unrooted phylogenetic tree using the full-length aligned DCL protein sequences by the maximum likelihood method. Our results showed that the unrooted phylogenetic tree from all the plant DCL protein sequences (273 sequences belonging to 73 species) supported via the bootstrap values most probably due to the short divergence (Fig. 1A). In this phylogenetic tree, the DCL family of proteins clustered into four main classes (DCL1, DCL2, DCL3, and DCL4 subgroups [33, 49]). Our phylogenetic result was in agreement with the previous classification of the plant DCLs subfamilies in terms of the tree topology. Rooting the global DCL tree using outgroup assigned polarity to the unrooted tree, which proposed the most likely evolutionary events happen after the divergence from their common ancestor. Based on the topology of the rooted tree, DCL proteins divide into two distinct main clades after diverging from the common ancestor (Fig. 1B). The first clade comprises the DCL1 proteins separating from the other DCL clades. However, the second clade comprised of two DCL homologous sequences sets (DCL2 and DCL3/DCL4 subgroups), which one of them further sub-divided into another two main subfamilies (DCL3 and DCL4 subgroups). All four-plant DCL type (DCL1–4) clades presented in either Eudicotyledons or Liliopsida. In addition, the tree revealed the evolutionary relationship within each clade. As expected, DCL subfamilies are distributed globally in a single clade, which could be due to the evolution of each subfamily from their common ancestor separately. Therefore, our phylogenetic analysis illustrates that each DCL subfamilies evolved independently in the monophyletic, followed in previous studies [49]. Eudicotyledons/Liliopsids DCL proteins’ separation does not observe in the main tree. The DCL proteins from Liliopsida grouped in the DCL subfamilies did not separate from the others, and its homologs in a specific sub-branch. This finding suggests that they conserved across the lineage. There were also strongly supported bootstrap values for some interior nodes of the tree due to high sequence similarities indicating a relatively well-supported phylogenetic tree reconstruction. The close observation on the phylogenetic tree strongly supports the independent expansion of Eudicotyledons and Liliopsida DCL proteins. Therefore, to generate a clear picture of how the independent development has occurred, we reconstructed the two monophyletic lineages as separate phylogenetic trees.

The constructed phylogenetic tree from Eudicotyledons species revealed four distinct subfamilies, similar to the branching structure of the phylogenetic tree reconstructed from the complete set of the higher plant species (Supplementary file 3). The evolutionary relationship within every subgroup follows the same pattern of the entire plant DCL protein sequences tree. Gene duplications are identified by searching for all branching points in the topology with at least one species present in both subtrees of the branching point (Supplementary file 4). Some sub-clades clearly illustrate orthologues relationships (derived by speciation) based on their branch distance agreement with the species tree as in the cases of *Prunus avium*, *P. persica*, and *P. mume* DCL1 proteins. Some clades reflect a recent gene duplication event. For example, we detected the DCL protein duplicated copy in *Camelina sativa*, *Medicago truncatula*, *Populous eupheratica*, *Nicotiana tomentoformis*. Sometimes, the gene duplication precedes the speciation (e.g., *Citrus clementina* from *Citrus sinensis* or divergence of *Solanum pennelii* and *S. tuberosum* from their common ancestor). Liliopsida DCL protein phylogenetic analysis revealed the same trend (Supplementary file 5). Also, close observation revealed that the Liliopsida DCL proteins tree topology is highly similar to the tree presented for Eudicotyledons. Our results suggest that the divergence of the DCL proteins in four main subgroups formed right before the split between the Eudicotyledons/Liliopsida lineages. The aggregation pattern of DCL proteins in the reconstructed trees showed that the DCL protein family in higher plants seem to share a common origin, and the main duplication events for the formation of subfamilies occurred before the Eudicotyledons/Liliopsida split. Therefore, the emergence of the four DCL subgroups can date back to before the derivation of these lineages, and they may have evolved independently from their ancestral DCL. Our results agree with Mukherjee et al. [33], who described that DCL proteins give rise to four distinct subgroups before or around the divergence of moss from higher plants. Our data revealed that after the Eudicotyledons/Liliopsida derivation, DCL proteins seem to undergo a similar evolutionary history before lineages separation. On the other hand, Mukherjee and colleagues identified the DCL1 and DCL3 moss and Selagaginella orthologues previously [33], which is the other evidence for the main plant DCLs origin. Moreover, MSA on DCL protein full-length sequences present a high similarity in the Eudicotyledons/Liliopsida monophyletic lineages, especially within the subfamilies (Data not shown but are available from the authors on request); inferring a high level of conservation.

### Analysis of conserved motifs and motif composition

Analysis of protein conserved motifs and their motif composition provides additional clues about the evolutionary relationship of the protein family. In Eudicotyledons, the motifs 1, 2, 3, 6, 9, 10, 15, and 20 represented the RNASE_3_2 Ribonuclease III family, 1 and 2 helicase C-terminus, RNASE_3_2 Ribonuclease III, RNASE_3_2 Ribonuclease III, Dicer dsRNA-binding fold, PAZ, and Dicer dsRNA-binding fold domains, respectively. However, the other motifs have not yet been characterized (Table 2). Similar trends resulted in Liliopsida species for DCL protein sequences. The motifs 1, 2, 3, 5, 7, 8, 9, and 11 maintain the specified domains. However, the others have not yet been determined (Table 3).

**Table 2:**
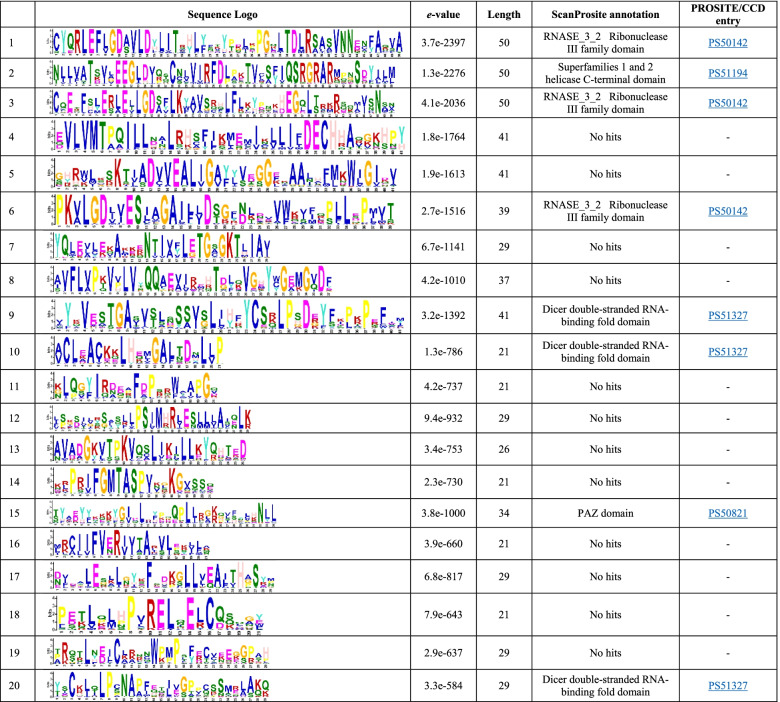
Conserved motifs identified in Eudicotyledons DCL proteins by MEME suite and their characteristics

**Table 3:**
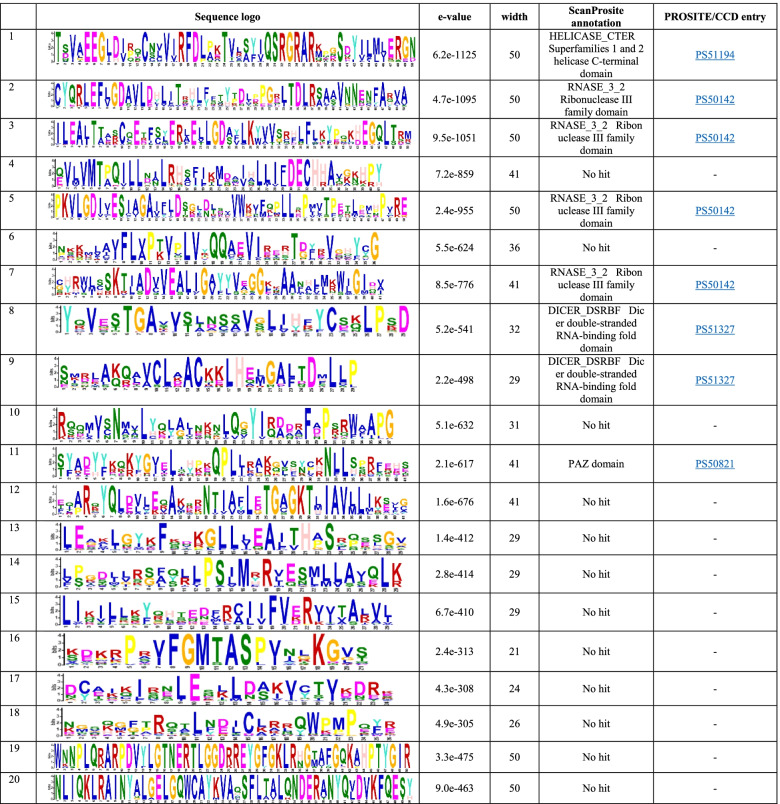
Conserved motifs identified in Liliopsida DCL proteins by MEME suite and their characteristics

The DCL sequence motifs in Liliopsida and Eudicotyledons were compared and found highly conserved. The motifs 1 and 4 in Eudicotyledons DCL sequences were the same as the motifs 2 and 4 in Liliopsida DCL protein sequences. Additionally, the motifs 5, 6, 9, 14, 15, and 17 in the Eudicotyledons DCL proteins sequence set seemed similar to 7, 5, 8, 16, 11, and 13 motifs within Liliopsida, suggesting their biological importance. To obtain more insights into the diversity of motif compositions, the motifs identified from each DCLs were aligned and compared from species of Liliopsida and Eudicotyledons (Data not shown but are available from the authors on request). The results were evidence of the conservation of critical residues in lineage-specific and inter lineage motifs. However, the spacing between their completely conserved residues could vary considerably (as shown in the motif sequence logo in Tables 2 and 3). In each motif, the fully conserved residues from the same geometry in alignment might be a signature of specific domains. Such key residues may be critical for their function, and mutation of some of these residues probably can alter the protein function and even be deleterious.

**Fig. 2:**
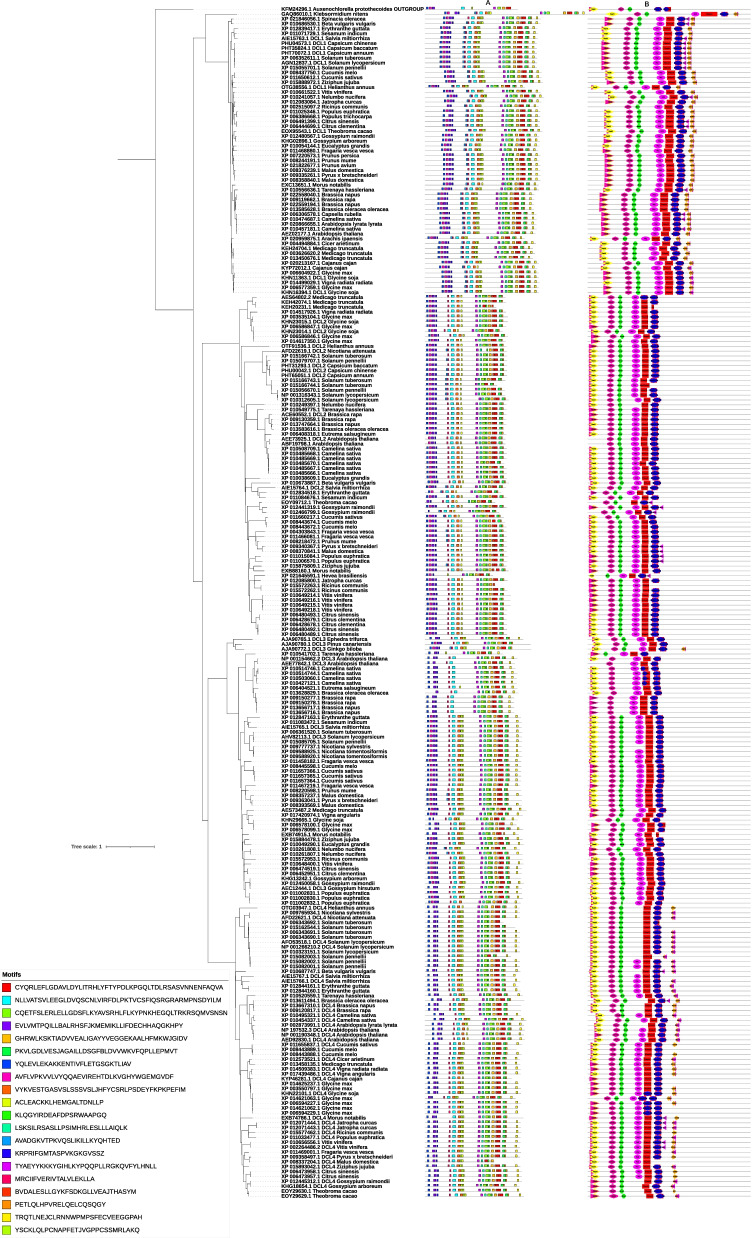
Graphical representation of the conserved motifs (**A**)/domains (**B**) variations and their architecture in Eudicotyledons DCL protein sequence and their evolutionary relationship within each DCL protein clade. The protein motifs were obtained using MEME suit (http://memesuite.org/ [1]) and the functional domains were predicted by querying the protein sequence in the Hmmscan search tool (https://www.ebi.ac.uk/Tools/hmmer/search/hmmscan [11]) against the Pfam database (http://pfam.xfam.org/). Different motifs are represented by different colored boxes and the consensus sequences of the motifs are listed. The domains are marked with different shapes and colors

**Fig. 3:**
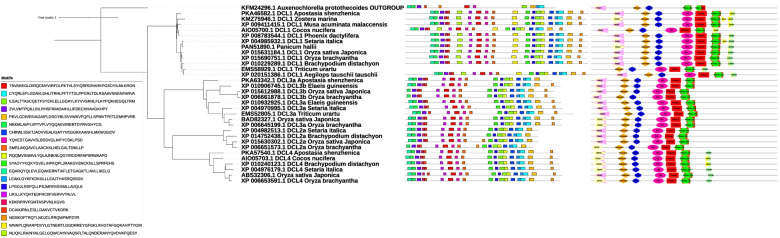
Graphical representation of the conserved motifs (**A**)/domains (**B**) variations and their architecture in Liliopsida DCL protein sequence and their evolutionary relationship within each DCL protein clade. The protein motifs were obtained using MEME suit (http://memesuite.org/ [1]) and the functional domains were predicted by querying the protein sequence in the Hmmscan search tool (https://www.ebi.ac.uk/Tools/hmmer/search/hmmscan [11]) against the Pfam database (http://pfam.xfam.org/). Different motifs are represented by different colored boxes and the consensus sequences of the motifs are listed. The domains are marked with different shapes and colors

All the DCLs in Eudicotyledons lineages harbor the conserved motifs 1, 2, 3, 6, 9, 10, and 15 suggests the presence of these domains to be quintessential for the functionality of this family (Fig. 2). They were common among *A. protothecoides*and the DCL proteins from Eudicotyledons/Liliopsida lineages (Fig. 3). We noticed that 1, 2, 3, 4, 5, 6, 8, 12, 13, 17, and 18 conserved motifs are common among *A. protothecoides* and Liliopsida DCLs. The motifs 1, 6, and 9 in Eudicotyledons and 2, 5, and 8 in Liliopsida were also detected among *A. protothecoides* rudimentary DCL form, indicating their deep conservation and importance; suggesting to have a common origin. The DCL1 clade members share the same conserved motifs. A similar motif compound offers the conserved role of the DCL1 proteins in the Eudicotyledons plant cells and their importance for their cellular function. DCL2 containing subgroups was predicted to lack the 13, 19, and 20 motifs. Our analysis indicated that the DCL2 clade members within the same subgroup exhibit similar motif composition revealed the relation to others. Analysis of motif conservation and motif composition in this clade indicated that they might derive from a common ancestor. Motif representation analysis within the DCL3/DCL4 clade revealed the DCL4 subgroup a similar structure in terms of motif composition and orientation, and motifs 8 and 13 were not detected in all the subgroup sequences. It is possibly indicative of some degree of functional conservation.

### Domain identification and architecture

Multi-domain proteins may exhibit more complex domain organization and architecture among the homologous sequences. Domain shuffling, intramolecular duplications, fusion and fission, novel domain acquisition, and its loss are the events that can cause some variations in the domain organization, i.e., both composition and orientation, creating independent domain combinations. In this sense, the protein sequences were searched against the Pfam to predict functional domains by Hmmscan. From N- to C-termini in Eudicotyledons DCL proteins, Ribonuclease III (PF00636.28), Dicer dimerization (PF03368.16), Ribonuclease 3-3 (PF14622.8), PAZ (PF02170.24), DEAD (PF00270.31), Helicase C (PF00271.33), dsRM (PF00035.28), ResIII (PF04851.17), and DND1-dsRM (PF14709.9) domains were extracted and analyzed (Supplementary file 6, A). We predicted similar domains in the Liliopsida DCL sequences set (Supplementary file 6, B). Our analysis showed that despite the long evolutionary course from the divergence of these two lineages, no critical variations were evident. In addition, our data were following earlier reports on the presence of Ribonuclease III (PF00636.28) and Ribonuclease 3-3 (PF14622.8) domains in Eudicotyledons DCL ([12, 48]; Fig. 2). Our results revealed that in all Eudicotyledons DCL proteins inspected, the DEAD (PF00270.31) domain was present, except for XP 021645591.1 sequence from *Hevea brasiliensis*. In most, if not all, of the Eudicotyledons DCLs, we identified the DND1-dsRM (PF14709.9), PAZ (PF02170.24), and Dicer dimerization (PF03368.16) domains. Domains were in the same combinations and order within the DCL1 subgroup, another reason for their conservation and evolution from their joint ancestor architecture (Fig. 2). Such localization seems significant for the DCL protein function. Members of the same subfamily have the same domain organization. As shown in Figs. 2 and 3, the DCL proteins in some branches have one or more domain gain and loss that makes them vary from those of the others. Domain gain and loss are frequent events in plants’ multi-domain proteins evolution [51].

The presence of Ribonuclease III (PF00636.28), Dicer dimerization (PF03368.16), Ribonuclease 3-3 (PF14622.8), PAZ (PF02170.24), DEAD (PF00270.31), Helicase-C (PF00271.33), dsRM (PF00035.28), ResIII (PF04851.17), and DND1-dsRM (PF14709.9) domains characterize the DCL1 subfamily in Eudicotyledons. In contrast to DCL1, the DCL2 subfamily lack dsRM (PF00035.28) and DND1-dsRM (PF14709.9) domains. However, in some DCL2 members, the dsRM domain (PF00035.28) was detected. In the DCL3 subfamily members, we identified a similar trend. A branch within the DCL3 subfamily lacks the Dicer dimerization (PF03368.16) domain, excepting other branches (Fig. 2). The DCL3 subfamily shares similar domain architectures with the DCL2 subfamily suggesting close biological function. Some branches of the plant DCL4 proteins have lost the PAZ (PF02170.24) domain. Such a situation may indicate diverse functions for the DCL4 lacking the PAZ domain (PF02170.24). Species with DCLs lacking the PAZ domain need to consider with caution as the data on their proteome might be incomplete. In the true cases, functional consequences of proteins lacking the PAZ domain remain to address.

Ribonuclease III (PF00636.28), PAZ (PF02170.24), Dicer dimerization (PF03368.16), Ribonuclease 3-3 (PF14622.8), Helicase C (PF00271.33), ResIII (PF04851.17), and DEAD (PF00270.31) were found in the Liliopsida DCL proteins as well (Fig. 3). However, DND1-dsRM (PF14709.9) and dsRM (PF00035.28) domains were the unpredicted domains in Liliopsids DCL2 and DCL3 subfamilies. In the DCL of *Klebsormidium nitens*, the PAZ (PF02170.24) domain-containing protein was detected, but not in *A. protothecoides*. These results suggest that the PAZ domain probably emerged de novo before the divergence of plants and *K. nitens* from their common ancestors from which the plants evolved. In general, our results support the hypothesis that the DCL protein subfamilies originated from the same ancestor before the divergence of the plant’s main monophyletic lineages. Therefore, these subfamilies of the DCLs existed before the split of monocot and dicot plants. Additionally, the deviation between different branches originated from Liliopsida and Eudicotyledons architecture seems to be due to the recombination and domain loss rather than de novo domain gain in their predecessor. These results may explain some functional overlaps among plant DCLs.

### Structural comparative analyses of DCL protein subfamilies—a case study

**Fig. 4:**
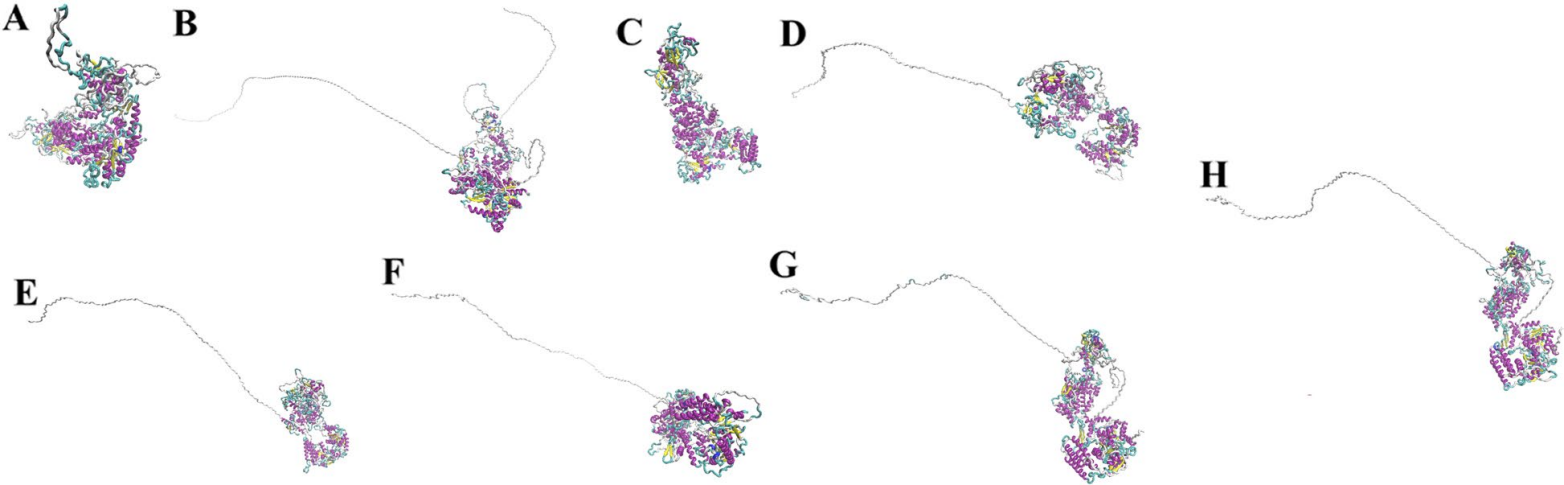
The presentation of predicted structure for DCL proteins encoded in *A. thaliana* and *A. protothecoides* by visualizing the predicted PDB files in the VMD protein visualization program. The proteins structure was modeled by searching their sequence in the HHpred search tool throughout the PDB and modeled by the MODELLER software. **A**: *A._protothecoides* DCL protein. **B**: *A. thaliana* DCL1 AE202177.1. **C**: *A. thaliana* DCL2 AEE73925.1. **D**: *A. thaliana* DCL3 AEE77842.1. **E**: *A. thaliana* DCL3 NP 001154662.2. **F**: *A. thaliana* DCL4 AED92830. **G**: *A. thaliana* DCL4 NP 001190348. **H**: *A. thaliana* DCL4 NP197532.3

Changes of single residues, insertions, deletions, and repetition due to the mutations are common in proteins. They accumulate over the evolution and likely produce unfunctional proteins or proteins with uncharacterized functions. The other conceivable situation would be mutations in the active zone(s) that may alter the molecular function [36]. Gene duplication, exon shuffling, or post-translational modifications may lead to circular permutations between the two homologous proteins, with or without the functional domain(s). Such rearrangements make a non-sequential sequence/structure alignment between the two homologous structures [3]. Therefore, between two homologous proteins, the structural similarity is an elaboration from a common ancestor rather than the result of the parallel evolution. If the proteins retain the same molecular function, they have resulted from a light structural deviation. In this study, we conducted comparative analyses of the DCL protein structure within and between *A. thaliana* and *A. protothecoides species that are* sister to each other with a common ancestor. In addition, we compared the protein structures of the DCL4 subfamilies containing PAZ domain with non-containing ones. HHpred webserver was employed to search a significant match with a protein of known folding in the PDB database. Protein structural homology was deduced from those of the most similar sequences. The HHpred allows the MODELLER software to build the atomic coordinates of the DCLs in PDB format from their string (Supplementary file 7; Fig. 4). We employed the distance matrix alignment (DALI) server for structural comparison and visualization superimposition of the predicted models. The structural similarity between protein structures and their structural relationship resulted from the dendrogram constructed by average linkage clustering of the structural similarity matrix based on the Dali score. The Dali structural similarity dendrogram showed that DCL protein homologs in *A. thaliana* belong to DCL subfamilies and are structurally related to others. In particular, the results indicated that the DCL structures diverged from a common structural ancestor with *A. protothecoides*.

**Fig. 5:**
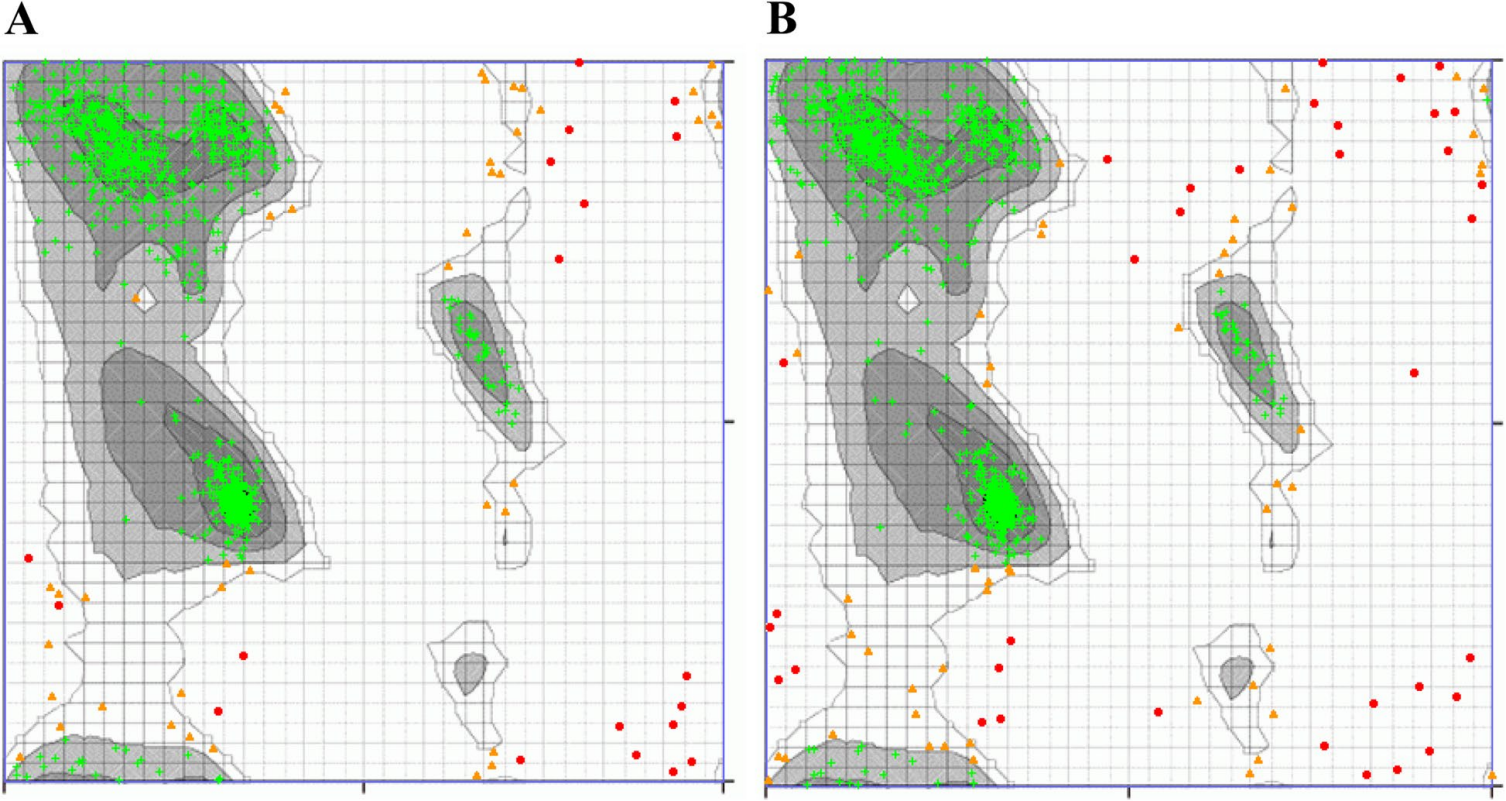
Ramachandran plot of **A**: PAZ domain-loss (*A. lyrata*; XP 002873991.1) and **B**: PAZ domain containing (*A. thaliana*; NP 197532.3) DCL protein. The plot calculation was done by uploading the PDB-predicted file to the Ramachandran plot server (https://zlab.umassmed.edu/bu/rama/)

We also considered all-against-all structural comparisons of the encoded DCL subfamilies in *A. thaliana*. The results indicated that the subfamily members were structurally similar (Table 4). It is noteworthy that DCL4 (NP197532.3 and AED92830) have the same structure. The result illustrated that the topmost similar structures were between DCL3 (NP001154662.2) and DCL4s (NP197532.3 and AED92830) with Dali *Z*-scores of 43.0 and RMSD = 10.8 Å. In addition, DCL1 (AE202177.1) showed the topmost similarity to the structure of *A. protothecoides* DCL. DCL4 (NP001190348) is a more distant structural relative to the *A. protothecoides* DCL. A consensus result of the structural similarity between DCLs among subfamilies proposed that they may have overlapping functions towards their dsRNA targets; results have already been reported elsewhere [12, 27]. The structural RMSD comparisons (the deviation between two superimposed atomic coordinates) of all-against-all of encoded DCL subfamilies showed high similarities. In the case of PAZ domain-containing (*A. thaliana*; NP 197532.3) and PAZ domain-lacking (*A. lyrata*; XP 002873991.1) structural comparison, RMSD of the Cα atomic coordinates were 4.3 Å with the estimated *Z*-score of 36.5. The sequence identity between these sequences was 84%, both structurally were similar. The structural similarity suggests that they diverged from a common structural ancestor. However, some deviance was evident in the PAZ domain because of the mutations occurring in the sequences during their evolution. Besides, it seems that such departure could not alter the protein and, or PAZ domain function. To clarify this point and the need for more evidence, we considered their Ramachandran plots were predicted by uploading their PDB-predicted file to the Ramachandran Plot server (https://zlab.umassmed.edu/bu/rama/; Fig. 5). These values for the respective selected DCLs were as follows: PAZ domain loss (95.673% in the favored region, 3.001% in the allowed area, and 1.326% in outlier region), PAZ domain-containing (93.942% in favored territory, 3.621% in allowed region, and 2.437% in outlier region). The plots provide an additional piece of evidence supporting the above hypotheses.

**Table 4:** Structural similarity (Dali *Z*-scores)/root mean squared deviation (RMSD) matrix of the protein homologs from DCL protein subfamilies in *A. thaliana*

	DCL2 AEE73925.1		***A. protothecoides***		DCL3 AEE77842.1		DCL4 NP001190348		DCL1 AE202177.1		DCL3 NP001154662.2		DCL4 NP197532.3		DCL4 AED92830	
	*Z* scores	RMSD (Å)	*Z* scores	RMSD (Å)	*Z* scores	RMSD (Å)	*Z* scores	RMSD (Å)	*Z* scores	RMSD (Å)	*Z* scores	RMSD (Å)	*Z* scores	RMSD (Å)	*Z* scores	RMSD (Å)
**DCL2 AEE73925.1**	60.1	0.0														
***Auxenochlorella protothecoides***	31.4	3.8	59.3	0.0												
**DCL3 AEE77842.1**	17.3	8.3	14.9	16.8	61.0	0.0										
**DCL4 NP001190348**	18.6	7.6	13.6	18.2	29.2	11.7	60.4	0.0								
**DCL1 AE202177.1**	21.4	13.4	16.8	17.0	30.8	25.6	35.7	28.6	62.1	0.0						
**DCL3 NP001154662.2**	19.5	8.1	15.6	19.8	39.0	6.8	36.9	16.8	40.4	18.5	59.1	0.0				
**DCL4 NP197532.3**	16.9	6.9	16.6	18.0	29.9	11.8	36.0	4.2	38.3	19.3	43.0	10.8	61.2	0.0		
**DCL4 AED92830**	16.9	6.9	16.6	18.0	29.9	11.6	36.0	4.2	38.3	19.1	43.0	10.8	61.2	0.0	61.2	0.0

## Conclusions

Small RNAs are essential mediators of gene expression in almost all eukaryotic lineages. They are involved in many biological processes, including but not limited to the development, organogenesis, and defense against genomic-invasive materials such as viruses and transposons, and in response to biotic and abiotic stresses. Several players and mediators are involved in long dsRNA precursors processing into mature small RNAs. However, Dicer or Dicer-like proteins are the key components, playing a pivotal role in small RNA biochemical processing and generation.

We aimed to study the plant Dicer evolutionary history, possible sequence, and structural relationships between DCL protein subfamilies in two plant monophyletic lineages. According to our finding, four distinct conserved DCL subfamilies are among the two plant monophyletic lines. Each DCL (i.e., DCL1-DCL4) distribute in their single clades after diverging from their common ancestor and before emerging into higher plants. Therefore, it seems that the main duplication events for the formation of the DCL subfamilies occurred before the Eudicotyledons/Liliopsida split and before the appearance of moss, and after the single-cell green algae. It seems that the expansion of the DCLs in Eudicotyledons and Liliopsida has happened, resulting in speciation possibilities rather than duplication. However, we found limited duplicating events for *DCL*s among the plant species. We also observed the same trends among the main DCL subfamilies from functional unit composition and architecture. Despite the long evolutionary course from the divergence of Liliopsida lineage from the Eudicotyledons, a significant diversifying force to domain composition and orientation was absent. Thus, huge functional variation is not expected. The results of this study provide a deeper insight into DCL protein evolutionary history and possible sequence and structural relationships between DCL protein subfamilies in the main higher plant monophyletic lineages; i.e., Eudicotyledons and Liliopsida.

## Supplementary Information

**Additional file 1. **List of the DCL protein sequences considered for this study.

**Additional file 2.** Multiple sequence alignment of plant DCL protein data set using Muscle with its default parameters.

**Additional file 3. **Evolutionary analysis of Eudicotyledons DCL proteins. The evolutionary history was inferred using the Maximum Likelihood method and JTT matrix-based model. The tree with the highest log likelihood (-10878.12) is shown. The percentage of trees in which the associated taxa clustered together is shown below the branches. Initial tree(s) for the heuristic search were obtained automatically by applying Neighbor-Joining and BioNJ algorithms to a matrix of pairwise distances estimated using the JTT model. The topology with superior log likelihood value was selected. A discrete Gamma distribution was used to model evolutionary rate differences among sites (2 categories (+*G*, parameter = 1.7788)). The tree is drawn to scale, with branch lengths measured in the number of substitutions per site. The analysis involved 243 polypeptide sequences of Eudicotyledons and *A. protothecoides* DCL protein sequences as outlier. All positions containing gaps and missing data were eliminated (complete deletion option). A total of 129 positions was identified in the final dataset. Evolutionary analyses were conducted in MEGA11 and visualized by iTOL v5 online tool.

**Additional file 4.** Gene duplications are identified by searching for all branching points in the topology of Eudicotyledons DCL proteins phylogenetic tree with at least one species being present in both subtrees of the branching point. Evolutionary analyses were conducted in MEGA11 and visualized by iTOL v5 online tool.

**Additional file 5. **Evolutionary analysis of Liliopsida DCL proteins. The evolutionary history was inferred by the Maximum Likelihood method and JTT matrix-based model. The tree with the highest log likelihood (-30151.26) is shown. The percentage of trees in which the associated taxa clustered together is shown below the branches. Initial tree(s) for the heuristic search were obtained automatically by applying Neighbor-Joining and BioNJ algorithms to a matrix of pairwise distances estimated using the JTT model, and then selecting the topology with superior log likelihood value. A discrete Gamma distribution was used to model evolutionary rate differences among sites (2 categories (+*G*, parameter = 2.8058)). The rate variation model allowed some sites to be evolutionarily invariable ([+*I*], 4.42% sites). The tree is drawn to scale, with branch lengths measured in the number of substitutions per site. This analysis involved 31 polypeptide sequences of Liliopsida and *A. protothecoides* DCL protein sequences as outlier. All positions containing gaps and missing data were eliminated (complete deletion option). There were 1017 positions in the final dataset. Evolutionary analyses were conducted in MEGA11 and visualized by iTOL v5 online tool.

**Additional file 6.** List of functional domains predicted in the Eudicotyledons (A) and Liliopsida DCL protein sequences by querying the protein sequence in Hmmscan search tool (https://www.ebi.ac.uk/Tools/hmmer/search/hmmscan [11];) against the Pfam database (http://pfam.xfam.org/) and their characteristics.

**Additional file 7. **The predicted DCL protein structures encoded in *A. thaliana* and *A. protothecoides* in PDB file format from their string by searching the known structure in the PDB database using HHpred search tool, and creating the PDB file by MODELLER.
