# Supplementary figures and images for: Comparative phylogeny and evolutionary analysis of Dicer-like protein family in two plant monophyletic lineages
Source: J Genet Eng Biotechnol. 2022 Jul 12;20:103. doi: 10.1186/s43141-022-00380-x (PMC9276914; doi:10.1186/s43141-022-00380-x)

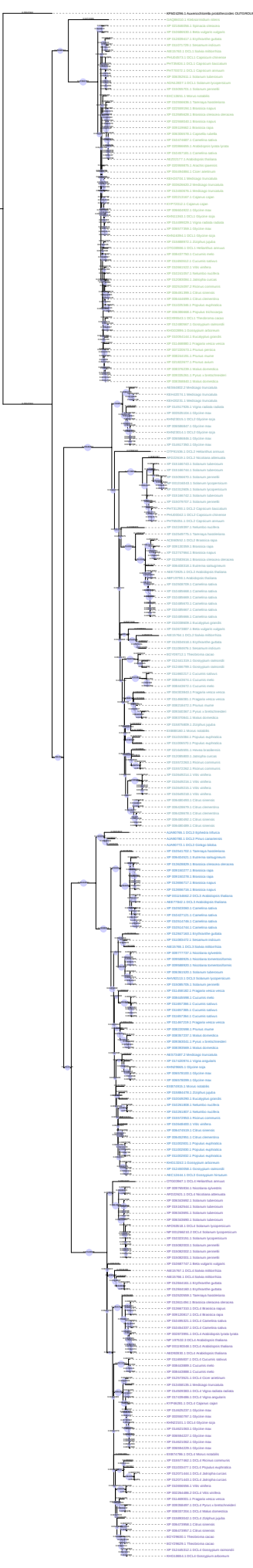

Supplement: Supplementary file 3 — Additional file 3. Evolutionary analysis of Eudicotyledons DCL proteins. The evolutionary history was inferred using the Maximum Likelihood method and JTT matrix-based model. The tree with the highest log likelihood (-10878.12) is shown. The percentage of trees in which the associated taxa clustered together is shown below the branches. Initial tree(s) for the heuristic search were obtained automatically by applying Neighbor-Joining and BioNJ algorithms to a matrix of pairwise distances estimated using the JTT model. The topology with superior log likelihood value was selected. A discrete Gamma distribution was used to model evolutionary rate differences among sites (2 categories (+G, parameter = 1.7788)). The tree is drawn to scale, with branch lengths measured in the number of substitutions per site. The analysis involved 243 polypeptide sequences of Eudicotyledons and A. protothecoides DCL protein sequences as outlier. All positions containing gaps and missing data were eliminated (complete deletion option). A total of 129 positions was identified in the final dataset. Evolutionary analyses were conducted in MEGA11 and visualized by iTOL v5 online tool. [file 43141_2022_380_MOESM3_ESM.pdf]

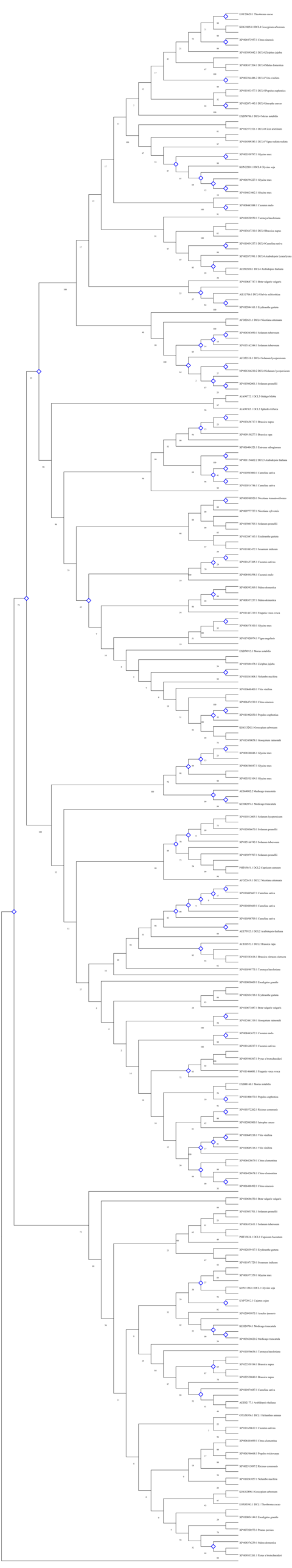

Supplement: Supplementary file 4 — Additional file 4. Gene duplications are identified by searching for all branching points in the topology of Eudicotyledons DCL proteins phylogenetic tree with at least one species being present in both subtrees of the branching point. Evolutionary analyses were conducted in MEGA11 and visualized by iTOL v5 online tool. [file 43141_2022_380_MOESM4_ESM.pdf]

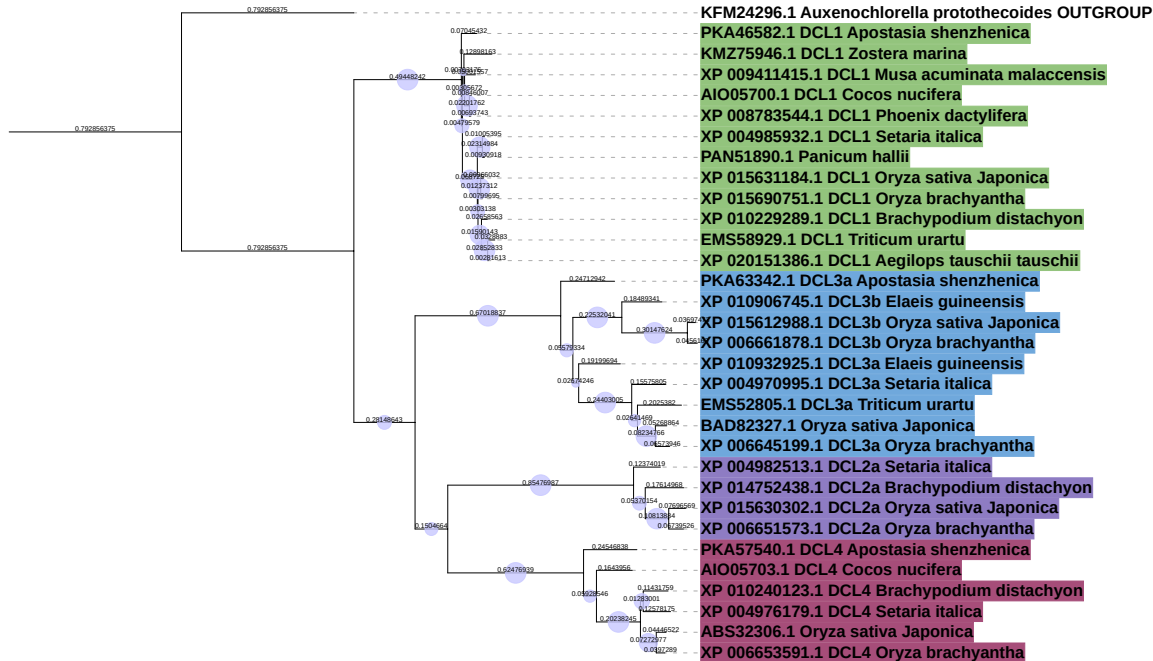

Supplement: Supplementary file 5 — Additional file 5. Evolutionary analysis of Liliopsida DCL proteins. The evolutionary history was inferred by the Maximum Likelihood method and JTT matrix-based model. The tree with the highest log likelihood (-30151.26) is shown. The percentage of trees in which the associated taxa clustered together is shown below the branches. Initial tree(s) for the heuristic search were obtained automatically by applying Neighbor-Joining and BioNJ algorithms to a matrix of pairwise distances estimated using the JTT model, and then selecting the topology with superior log likelihood value. A discrete Gamma distribution was used to model evolutionary rate differences among sites (2 categories (+G, parameter = 2.8058)). The rate variation model allowed some sites to be evolutionarily invariable ([+I], 4.42% sites). The tree is drawn to scale, with branch lengths measured in the number of substitutions per site. This analysis involved 31 polypeptide sequences of Liliopsida and A. protothecoides DCL protein sequences as outlier. All positions containing gaps and missing data were eliminated (complete deletion option). There were 1017 positions in the final dataset. Evolutionary analyses were conducted in MEGA11 and visualized by iTOL v5 online tool. [file 43141_2022_380_MOESM5_ESM.pdf]
